# Self-perception of stuttering: association with self-perception of hearing, fluency profile, and contextual aspects

**DOI:** 10.1590/2317-1782/e20240103en

**Published:** 2025-01-20

**Authors:** Camila Eduarda Elias Silva, Denise Brandão de Oliveira Britto, Stela Maris Aguiar Lemos

**Affiliations:** 1 Programa de Pós-graduação em Ciências Fonoaudiológicas, Universidade Federal de Minas Gerais – UFMG - Belo Horizonte (MG), Brasil.; 2 Departamento de Fonoaudiologia, Universidade Federal de Minas Gerais – UFMG - Belo Horizonte (MG), Brasil.

**Keywords:** Stuttering, Adult, Quality of Life, Fluency, Auditory Skills

## Abstract

**Purpose:**

This study investigated the association between self-perception of stuttering and self-perception of hearing, speech fluency profile, and contextual aspects in Brazilian adults who stutter.

**Methods:**

Fifty-five adults who stutter (ages 18 to 58 years), speakers of Brazilian Portuguese speakers, participated in an observational study that included: (a) a clinical history survey to collect identification, sociodemographic, clinical, and assistance data; (b) the Brazil Economic Classification Criteria (CCEB); (c) a hearing self-perception questionnaire (Speech, Spatial and Qualities of Hearing Scale – SSQ, version 5.6); (d) self-perception of the impact of stuttering (Brazilian Portuguese version of the Overall Assessment of the Speaker's Experience of Stuttering – Adults – OASES-A); and (e) an assessment of speech fluency (Fluency Profile Assessment Protocol -- PAPF). Data analysis consisted of descriptive and bivariate analysis using Pearson’s chi-square, Mann-Whitney, Kruskal-Wallis, and Spearman correlation coefficient.

**Results:**

Most participants were classified as moderate to severe in the total classification of the impact of stuttering. There were moderate and weak negative correlations between the participants' self-perception of stuttering and self-perception of hearing.

**Conclusion:**

Self-perception of auditory abilities was greater to the extent that self-perception of the impacts of stuttering on quality of life was lower.

## INTRODUCTION

Stuttering is a communication disorder with various manifestations that can negatively affect a person’s quality of life^(1-3)^. Its etiology encompasses multidimensional aspects involving neurobiological, genetic, environmental, auditory, linguistic, behavioral, and social factors.

Various theories on the etiology of stuttering have been proposed, but its specific neurobiological cause and origin remain uncertain^(4)^. Brain mapping studies^(5,6)^ have demonstrated that the brains of people who stutter have structural and functional differences, compared to the brains of people who do not stutter. These differences are often noted in areas related to the processing of auditory information, including hypoactivity in the superior temporal gyrus and cognitive mechanisms involved in the auditory perception of adults who stutter^(7)^.

Auditory perception involves cognitive processes such as working memory and attentional mechanisms. Problems involving these cognitive processes have been identified in people who stutter^(8)^. A comparative study^(6)^ assessing electrophysiological measures (mismatch negativity and P300) showed differences in behavioral and electrophysiological measures between people who stutter and do not stutter, suggesting that the lower amplitude in mismatch negativity and P300 tests can indicate auditory processing deficits. Deficits in working memory and attention may cause auditory processing difficulties, which may, in turn, affect speech fluency.

According to the Diagnostic and Statistical Manual of Mental Disorders-5 (DSM-5), stuttering encompasses not only fluency behaviors (prolongations, blocking, and syllable, sound, and word repetitions) and other manifestations but also intrinsic factors such as behavioral, emotional, and cognitive reactions^(9)^. This broader DSM-5 definition considers stuttering as a disorder that causes “anxiety or limitations in effective communication, social participation, and academic or occupational performance”. The adverse impacts of stuttering on a person’s life who stutter are related to the difficulty they face daily in saying what they want, causing affective, behavioral and cognitive reactions. These reactions can be exacerbated by outside environmental factors, such as the reactions of listeners^(10)^, justifying the importance of understanding stuttering based on the premises of the International Classification of Functioning Disability and Health (ICF).

The Overall Assessment of the Speaker’s Experience of Stuttering – Adults (OASES-A) it is a instruments widely used in the field^(10-14)^, which describes stuttering based on the experience of the person who stutters^(4)^, and it is based on the ICF. Many researchers^(10-18)^ have studied stuttering based on the self-perception of people who stutter to better understand the implications of the disorder and its relationship with other manifestations. Beilby^(19)^ highlights the relevance of research supported by the perception of people who stutter. The amount of stuttering observed by a listener is not necessarily related to the experience of adverse impact as perceived by the speaker. Thus, the manifestations depend on their unique characteristics and experiences^(20)^.

Understanding clinical aspects and other associated factors in the manifestation of stuttering based on the self-perception of the impacts of stuttering on the speaker’s quality of life may help better understand the disorder and establish efficient and lasting therapeutic procedures. Given the above, this study aimed to investigate the association between the self-perception of stuttering and of hearing, the speech fluency profile, and contextual aspects of people with self-perceived stuttering.

## METHODS

Participants were 55 adults who self-reported to be individuals who stutter, ages 18 to 58 years, whose mean age was 30.42±10.20 years and a median 27.00. All were speakers of Brazilian Portuguese. Participants were recruited through invitations with a subscription link developed in Google Forms, announced in support groups for people who stutter, websites, social media, institutions, and associations all over Brazil. They were contacted via the phone numbers or e-mails informed in the subscription to send them the informed consent form.

Most of participants were females (61.8%); most were high school graduates (56.4%) belonging to class B2 in CCEB (32.7%); and approximately half were employed (49.1%). Most subjects reported a family history of stuttering (72.7%), with onset in childhood (80.0%). Nearly all reported that they had previously received treatment for stuttering (90.9%).

The study excluded potential participants who did not produce a speech sample of at least 200 fluent syllables^(21)^, who did not complete all research instruments, who had a diagnosis or history of hearing loss or used hearing aids, or who had other diagnoses (e.g., attention-deficit/hyperactivity disorder) or sequelae of traumatic brain injury or stroke. The initial sample had 60 participants, but five were excluded – one for not meeting all inclusion criteria, and four for hearing loss in prior testing via pure-tone threshold audiometry. All participants were informed about the research objectives and procedures, agreed to participate, and signed an informed consent form. The research was approved by the institution’s Ethics Research Committee under evaluation report no. 4.532.878.

Data were collected from August 2021 to June 2022, both synchronously and asynchronously in a virtual setting^(22)^. Participants were initially interviewed in approximately 50-minute individual remote synchronous sessions on the Zoom platform. During this session, speech samples were collected, and participants completed the Speech, Spatial and Qualities of Hearing Scale (SSQ, version 5.6)^(23)^.

Hearing was self-assessed with SSQ^(23)^, which verifies the subjective experience and quantifies the inability to hear in realistic communication situations. SSQ version 5.6 has 49 questions divided into three parts – 14 of them address speech hearing, 17 investigate different spatial hearing components, and 18 approach the qualities of hearing. It is administered in interviews, and subjects score from 0 to 10 on their performance in each situation described in the items. Participants in this study were instructed to ascribe a value from 0 to 10 to the situation described by the researcher – 10 meant they were perfectly able to do what the question described, and 0 meant they were unable to do it. If the question referred to a situation that was not part of the person’s everyday life, they should answer “not applicable”.

The speech fluency profile was measured with the Fluency Profile Assessment Protocol (PAPF, in Portuguese)^(24)^, which provides information on disfluency quantitative and qualitative analyses based on audio and video recorded spontaneous speech samples. It classifies disfluencies (speech disruptions) into 12 categories, of which six are considered non-stuttered, and the other six are considered atypical or stuttering-like. The instrument calculates the speech speed (emission rate) – words per minute (information production rate) and syllables per minute (articulation speed rate) – and the frequency of disfluencies, characterizing the percentages of disrupted speech and stuttering-like disfluencies.

Sociodemographic data to characterize the sample (age, sex, educational attainment, and occupation) and their history of stuttering (heredity and time of onset) and assistance (previous speech therapy) were collected through individual medical history survey in the institution’s Speech, Language and Hearing Sciences outpatient center. For analysis, data on educational attainment and age were grouped and classified as illiterate, incomplete middle school, complete middle school, incomplete high school, high school graduate, incomplete higher education, and bachelor’s degree. Their ages were classified based on the median (27 years old) – up to 27 years old and 28 or more years old.

Data on the time of stuttering onset were grouped for analysis considering onset in childhood (up to 9 years and 11 months old), adolescence (from 10 years to 17 years and 11 months old), and adulthood (18 or more years old). Data on the participant’s occupations were grouped according to the National Occupation-Position Classification used by the Brazilian Institute of Geography and Statistics (IBGE)^(25)^, as follows: student, employed, domestic worker, self-employed, unpaid worker, and unemployed.

The Brazilian Economic Classification Criteria (CCEB 2021)^(26)^ and the Overall Assessment of the Speaker’s Experience of Stuttering – Adults (OASES-A)^(15)^ was answered on a questionnaire developed in Google Forms, to which a link was sent to participants via WhatsApp.

The CCEB 2021^(26)^ surveys the householder’s educational attainment and household characteristics, such as the presence and number of appliances, using statistical methods to define broad classes and segment them per purchasing power. For data analysis, the CCEB classes were grouped as follows: A/B1/B2 = A/B; C1/C2/D-E = C/D-E.

Self-perceived stuttering was measured and classified with OASES-A^(15)^, which verifies the degree of the adverse impact associated with stuttering on the participant’s quality of life. It has 100 items, organized into four different areas: general information, reactions to stuttering, communication in daily situations, and quality of life. All section items have Likert-type answers ranging from 1 to 5, whose scores indicate the impact of stuttering on various aspects of the speaker’s life. The scores are classified as mild, mild to moderate, moderate, moderate to severe, and severe. Besides the score and classification in each of the four parts, the test calculates the total score and classification of the effect of stuttering on the speaker’s quality of life. The classifications were grouped, because the dispersion of the distribution of participants in each category, as follows: Mild and Mild to Moderate = Mild/Moderate; Moderate and Moderate to Severe = Moderate/Severe; and Severe = Severe.

The response variable was the self-perceived effect of stuttering on the quality of life (total score and classification), while the explanatory variables were the self-perceived hearing, sex, age, educational attainment, occupation, socioeconomic classification, clinical-assistance factors related to their history of stuttering (heredity, time of onset, and SLH therapy), and fluency profile (percentage of speech disruption, percentage of stuttering-like disfluencies, number of normal disfluencies, number of stuttering-like disfluencies, and words per minute and syllables per minute rates).

To achieve the study objective, data were submitted to descriptive analysis through the frequency distribution of the categorical variables and analysis of the measures of central tendency and dispersion of the continuous variables. Association analyses were performed with Pearson’s chi-square and Kruskal-Wallis tests. The Kruskal-Wallis nonparametric test was used because the variables did not have a normal distribution, verified with the Shapiro-Wilk and Kolmogorov-Smirnov tests, whose values were lower than 0.05. In the case of statistical significance (i.e., p ≤ 0.05) in the Kruskal-Wallis test results, the Nemenyi multiple comparisons test was used to identify the pairs whose associations had been significant (p ≤ 0.05). The correlation analysis was performed through Spearman’s correlation coefficient, whose correlation magnitude was measured with the following parameters: weak = 0.0-0.4; moderate = 0.4-0.7; strong = 0.7-1.0; as long as the individual p-value ≤ 0.05^(27)^. Data were entered, processed, and analyzed in SPSS software, version 25.0.

## RESULTS

The results of the speech fluency profile and the measures of central tendency and dispersion of these variables and the scores of the effect of stuttering in each part of OASES-A are shown in Table 1.

**Table 1:** Descriptive measures of the profiles of speech fluency and scores of the impacts of stuttering on the quality of life

Variables	N	Mean	SD	Median	Minimum	Q_1_	Q_3_	Maximum
Non-stuttered disfluencies	55	12.40	7.71	11.00	3.00	8.00	15.00	44.00
stuttering-like disfluencies	55	14.33	20.17	11.00	0.00	5.00	18.00	147.00
Words per minute	55	97.69	23.78	94.30	23.00	84.60	109.60	156.00
Syllables per minute	55	187.27	47.08	185.40	45.00	160.00	210.50	291.00
Speech disruption (%)	55	13.13	12.14	10.90	3.00	7.40	15.80	93.00
Stuttering-like disfluencies (%)	55	7.03	9.80	5.30	0.00	2.50	8.90	71.00
Impact score – Section 1	55	2.93	0.54	3.10	2.00	2.45	3.35	4.00
Impact score – Section 2	55	3.21	0.73	3.13	2.00	2.73	3.83	5.00
Impact score – Section 3	55	2.99	0.72	2.88	1.00	2.52	3.48	5.00
Impact score – Section 4	55	3.16	0.94	3.17	1.00	2.44	3.80	5.00
Impact score – Total	55	3.06	0.64	3.10	2.00	2.53	3.55	4.00

Caption: N = number of individuals; SD = standard deviation; Q = quartile; % = percentage

The OASES-A scores showed that most subjects’ impacts of stuttering on the quality of life were classified as moderate to severe in Section 1 – General Information (45.5%), moderate and moderate to severe in Section 2 – Reactions to Stuttering (30.9% each), moderate in Section 3 – Communication in Daily Situations (36.4%), moderate to severe in Section 4 – Quality of Life, and moderate to severe in the total classification (40.0%).

The descriptive measures of the questions (Q) of self-perceived hearing (SSQ) part 1 (Speech Hearing), part 2 (Spatial Hearing) and part 3 (Qualities of Hearing) are shown in Table 2.

**Table 2:** Descriptive measures of the self-perceived hearing (SSQ) for questions - Parts 1, 2 and 3

Variables	N	Mean	SD	Median	Minimum	Q_1_	Q_3_	Maximum
SSQP1_Q1	55	7.84	2.49	8.00	0.00	6.00	10.00	10.00
SSQP1_Q2	55	9.71	0.71	10.00	6.00	10.00	10.00	10.00
SSQP1_Q3	55	8.15	2.21	9.00	3.00	7.00	10.00	10.00
SSQP1_Q4	55	6.80	2.59	7.00	1.00	5.00	9.00	10.00
SSQP1_Q5	55	8.29	1.73	9.00	3.00	7.00	10.00	10.00
SSQP1_Q6	55	5.87	2.88	7.00	0.00	4.00	8.00	10.00
SSQP1_Q7	53	7.38	2.10	8.00	2.00	6.00	9.00	10.00
SSQP1_Q8	52	6.83	2.53	7.00	1.00	5.00	9.00	10.00
SSQP1_Q9	55	7.13	2.58	8.00	0.00	5.00	9.00	10.00
SSQP1_Q10	55	4.64	2.95	5.00	0.00	3.00	7.00	10.00
SSQP1_Q11	55	6.58	2.37	7.00	1.00	4.00	8.00	10.00
SSQP1_Q12	55	7.60	1.99	8.00	3.00	6.00	10.00	10.00
SSQP1_Q13	55	7.04	3.02	7.00	0.00	5.00	10.00	10.00
SSQP1_Q14	55	4.64	3.00	5.00	0.00	2.00	7.00	10.00
SSQP2_Q1	54	7.00	2.84	8.00	0.00	5.00	9.25	10.00
SSQP2_Q2	55	7.53	2.67	8.00	0.00	6.00	10.00	10.00
SSQP2_Q3	55	9.13	1.55	10.00	3.00	9.00	10.00	10.00
SSQP2_Q4	55	8.09	2.34	9.00	0.00	7.00	10.00	10.00
SSQP2_Q5	53	6.89	2.52	7.00	0.00	5.50	9.00	10.00
SSQP2_Q6	55	7.85	2.40	9.00	0.00	7.00	10.00	10.00
SSQP2_Q7	55	7.55	2.38	8.00	0.00	6.00	10.00	10.00
SSQP2_Q8	54	7.48	1.94	8.00	1.00	7.00	9.00	10.00
SSQP2_Q9	55	7.60	2.29	8.00	0.00	7.00	9.00	10.00
SSQP2_Q10	52	6.65	2.94	8.00	0.00	5.00	9.00	10.00
SSQP2_Q11	53	6.79	2.74	7.00	0.00	5.00	9.00	10.00
SSQP2_Q12	55	7.67	2.37	8.00	0.00	7.00	10.00	10.00
SSQP2_Q13	55	7.93	2.19	8.00	0.00	7.00	10.00	10.00
SSQP2_Q14	53	8.09	3.07	10.00	0.00	6.50	10.00	10.00
SSQP2_Q15	51	6.94	2.87	7.00	0.00	5.00	10.00	10.00
SSQP2_Q16	51	7.39	2.65	8.00	0.00	5.00	10.00	10.00
SSQP2_Q17	54	7.81	2.27	8.00	0.00	6.00	10.00	10.00
SSQP3_Q1	55	8.44	2.56	10.00	0.00	8.00	10.00	10.00
SSQP3_Q2	55	7.55	3.32	9.00	0.00	5.00	10.00	10.00
SSQP3_Q3	55	8.67	2.29	10.00	0.00	8.00	10.00	10.00
SSQP3_Q4	55	9.02	1.97	10.00	1.00	9.00	10.00	10.00
SSQP3_Q5	55	9.15	1.45	10.00	4.00	9.00	10.00	10.00
SSQP3_Q6	55	9.36	1.79	10.00	0.00	10.00	10.00	10.00
SSQP3_Q7	55	6.93	3.07	8.00	0.00	5.00	10.00	10.00
SSQP3_Q8	55	9.27	1.27	10.00	5.00	9.00	10.00	10.00
SSQP3_Q9	54	9.11	1.71	10.00	3.00	9.00	10.00	10.00
SSQP3_Q10	55	9.04	1.60	10.00	3.00	8.00	10.00	10.00
SSQP3_Q11	55	9.05	2.04	10.00	0.00	9.00	10.00	10.00
SSQP3_Q12	55	8.04	2.63	10.00	0.00	7.00	10.00	10.00
SSQP3_Q13	55	8.00	2.43	9.00	0.00	7.00	10.00	10.00
SSQP3_Q14	55	6.47	3.20	7.00	0.00	4.00	10.00	10.00
SSQP3_Q15	55	7.27	2.93	8.00	0.00	5.00	10.00	10.00
SSQP3_Q16	43	7.84	2.91	9.00	0.00	6.00	10.00	10.00
SSQP3_Q17	55	9.20	1.65	10.00	0.00	9.00	10.00	10.00
SSQP3_Q18	55	5.78	3.13	6.00	0.00	4.00	8.00	10.00

Caption: N = number of individuals; SD = standard deviation; Q = quartile

The association analysis between the total classification of the impacts of stuttering and the sociodemographic data (sex, educational attainment, and occupation) and clinical data (family history, time of onset, and previous SLH therapy) showed a statistically significant association only between the total classification of the impacts of stuttering and occupation (p = 0.030) (Table 3).

**Table 3:** Association between OASES-A score (impacts of stuttering) and sociodemographic and clinical data

Variables	Impact of stuttering – Total			p-value
	Mild/Moderate N (%)	Moderate/Severe N (%)	Severe N (%)	
**Sex**				
Females	4 (80.0)	25 (61.0)	5 (55.6)	0.650
Males	1 (20.00	16 (39.0)	4 (44.4)	
Total	5 (100.0)	41 (100.0)	9 (100.0)	
**Educational attainment**				
High school graduate	1 (20.0)	25 (61.0)	5 (55.6)	0.332
Higher education incomplete	0 (0.0)	2 (4.9)	1 (11.1)	
Bachelor’s degree	4 (80.0)	14 (34.1)	3 (33.3)	
Total	5 (100.0)	41 (100.0)	9 (100.0)	
**Occupation**				
Student	0 (0.0)	18 (43.9)	3 (33.3)	0.030*
Employed	4 (80.0)	18 (43.9)	5 (55.6)	
Domestic worker	0 (0.0)	1 (2.4)	0 (0.0)	
Self-employed	0 (0.00	4 (9.8)	0 (0.0)	
Unpaid worker	1 (20.0)	0 (0.0)	0 (0.0)	
Unemployed	0 (0.0)	0 (0.0)	1 (11.1)	
Total	5 (100.0)	41 (100.0)	9 (100.0)	
**Family history**				
No	2 (40.0)	10 (24.4)	3 (33.3)	0.688
Yes	3 (60.0)	31 (75.6)	6 (66.7)	
Total	5 (100.0)	41 (100.0)	9 (100.0)	
**Time of onset**				
Childhood	5 (100.0)	34 (82.9)	5 (55.6)	0.105
Adolescence	0 (0.0)	6 (14.6)	2 (22.2)	
Adulthood	0 (0.0)	1 (2.5)	2 (22.2)	
Total	5 (100.0)	41 (100.0)	9 (100.0)	
**Previous SLH therapy**				
No	0 (0.0)	3 (7.3)	2 (22.2)	0.282
Yes	5 (100.0)	38 (92.7)	7 (77.8)	
Total	5 (100.0)	41 (100.0)	9 (100.0)	

Pearson’s chi-square test

* p-value ≤ 0.05

Caption: N = number of individuals; SLH = speech-language-hearing

The association between the total classification of the impacts of stuttering and sociodemographic data (age and CCEB) and speech fluency profile did not have statistically significant results.

The association between the total classification of the impacts of stuttering and self-perceived hearing with the Kruskal-Wallis test indicated a statistically significant association only in part 1 (Speech Hearing), question 14, which refers to the ability to talk to someone on the phone and someone else near them at the same time (p = 0.019). The Nemenyi test verified that the difference was between the mild/moderate and severe classifications (p = 0.015), with a higher median in the mild/moderate one. The analysis of parts 2 and 3 revealed no statistical significance in any of the items. The correlation analysis between the total score of the impact of stuttering and age, CCEB, and fluency profile revealed no statistically significant correlations (Table 4).

**Table 4:** Correlation between OASES-A scores (impacts of stuttering) and sociodemographic data and fluency profile

Variables	OASES-A	p-value
Age	-0.082	0.552
CCEB score	-0.129	0.358
Normal disfluencies	-0.115	0.405
Stuttering-like disfluencies	0.052	0.707
Words per minute	-0.049	0.720
Syllables per minute	-0.007	0.959
Percentage of speech disruptions	0.009	0.948
Percentage of stuttering-like disfluencies	0.053	0.700

Spearman coefficient

The correlation analysis between the total score of the impacts of stuttering and part 1 (Speech Hearing) of self-perceived hearing (SSQ) revealed statistically significant weak negative correlations with Q3 (-0.274), Q4 (-0.269), Q10 (-0.315), and Q12 (-0.366), and moderate ones with Q8 (-0.524), Q9 (-0.513), and Q11 (-0.424). In part 2 (Spatial Hearing) of self-perceived hearing (SSQ) did not find statistically significant correlations in any of the items. In part 3 (Qualities of Hearing), the correlation analysis – likewise with the Spearman correlation coefficient – revealed a statistically significant weak negative correlation between the total effect of stuttering and question 16 (0.380) (Table 5).

**Table 5:** Correlation between OASES-A total score (impact of stuttering) and self-perceived hearing (SSQ)

SSQ - Part 1 (Speech Hearing)	OASES-A		p-value
**SSQP1_Q1 –** You are talking with one other person and there is a TV on in the same room. Without turning the TV down, can you follow what the person you’re talking to says?	-0.123		0.370
**SSQP1_Q2 –** You are talking with one other person in a quiet, carpeted lounge-room. Can you follow what the other person says?	-0.136		0.321
**SSQP1_Q3 –** You are in a group of about five people, sitting round a table. It is an otherwise quiet place. You can see everyone else in the group. Can you follow the conversation?	-0.274*		≤0.001*
**SSQP1_Q4** - You are in a group of about five people in a busy restaurant. You can see everyone else in the group. Can you follow the conversation?	-0.269*		0.047*
**SSQP1_Q5 –** You are talking with one other person. There is continuous background noise, such as a fan or running water. Can you follow what the person says?	-0.086		0.535
**SSQP1_Q6 –** You are in a group of about five people in a busy restaurant. You CANNOT see everyone else in the group. Can you follow the conversation?	0.019		0.892
**SSQP1_Q7 -** You are talking to someone in a place where there are a lot of echoes, such as a church or railway terminus building. Can you follow what the other person says?	-0.137		0.329
**SSQP1_Q8 -** Can you have a conversation with someone when another person is speaking whose voice is the same pitch as the person you’re talking to?	-0.524*		≤0.001*
**SSQP1_Q9 -** Can you have a conversation with someone when another person is speaking whose voice is different in pitch from the person you’re talking to?	-0.513*		≤0.001*
**SSQP1_Q10 –** You are listening to someone talking to you, while at the same time trying to follow the news on TV. Can you follow what both people are saying?	-0.315*		0.019*
**SSQP1_Q11 –** You are in conversation with one person in a room where there are many other people talking. Can you follow what the person you are talking to is saying?	-0.424*		0.001*
**SSQP1_Q12 -** You are with a group and the conversation switches from one person to another. Can you easily follow the conversation without missing the start of what each new speaker is saying?	-0.366*		0.006*
**SSQP1_Q13 –** Can you easily have a conversation on the telephone?	-0.243		0.074
**SSQP1_Q14 –** You are listening to someone on the telephone and someone next to you starts talking. Can you follow what’s being said by both speakers?	-0.447*		0.010*
SSQ – Part 2 (Spatial Hearing)	OASES-A		p-value
**SSQP2_Q1 –** You are outdoors in an unfamiliar place. You hear someone using a lawnmower. You can’t see where they are. Can you tell right away where the sound is coming from?	0.091		0.513
**SSQP2_Q2 –** You are sitting around a table or at a meeting with several people. You can’t see everyone. Can you tell where any person is as soon as they start speaking?	-0.117		0.396
**SSQP2_Q3 –** You are sitting in between two people. One of them starts to speak. Can you tell right away whether it is the person on your left or your right, without having to look?	-0.092		0.502
**SSQP2_Q4 -** You are in an unfamiliar house. It is quiet. You hear a door slam. Can you tell right away where that sound came from?	-0.166		0.226
**SSQP2_Q5 –** You are in the stairwell of a building with floors above and below you. You can hear sounds from another floor. Can you readily tell where the sound is coming from?	-0.092		0.510
**SSQP2_Q6 -** You are outside. A dog barks loudly. Can you tell immediately where it is, without having to look?	-0.084		0.540
**SSQP2_Q7** - You are standing on the footpath of a busy street. Can you hear right away which direction a bus or truck is coming from before you see it?	-0.044		0.752
**SSQP2_Q8 –** In the street, can you tell how far away someone is, from the sound of their voice or footsteps?	-0.045		0.748
**SSQP2_Q9 -** Can you tell how far away a bus or a truck is, from the sound?	-0.054		0.698
**SSQP2_Q10 -** Can you tell from the sound which direction a bus or truck is moving, for example, from your left to your right or right to left?	0.098		0.491
**SSQP2_Q11 -** Can you tell from the sound of their voice or footsteps which direction a person is moving, for example, from your left to your right or right to left?	0.054		0.701
**SSQP2_Q12 -** Can you tell from their voice or footsteps whether the person is coming towards you or going away?	0.188		0.188
**SSQP2_Q13 -** Can you tell from the sound whether a bus or truck is coming towards you or going away?	0.140		0.140
**SSQP2_Q14 –** Do the sounds of things you are able to hear seem to be inside your head rather than out there in the world?	-0.065		0.643
**SSQP2_Q15 –** Do the sounds of people or things you hear, but cannot see at first, turn out to be closer than expected when you do see them?	0.249		0.088
**SSQP2_Q16 -** Do the sounds of people or things you hear, but cannot see at first, turn out to be further away than expected when you do see them?	-0.024		0.870
**SSQP2_Q17 -** Do you have the impression of sounds being exactly where you would expect them to be?	0.148		0.286
SSQ – Part 3 (Qualities of Hearing )		OASES-A	p-value
**SSQP3_Q1 –** Think of when you hear two things at once, for example, water running into a basin and, at the same time, a radio playing. Do you have the impression of these as sounding separate from each other?		0.052	0.708
**SSQP3_Q2 –** When you hear more than one sound at a time, do you have the impression that it seems like a single jumbled sound?		-0.014	0.922
**SSQP3_Q3 –** You are in a room and there is music on the radio. Someone else in the room is talking. Can you hear the voice as something separate from the music?		-0.210	0.123
**SSQP3_Q4 –** Do you find it easy to recognise different people you know by the sound of each one’s voice?		-0.045	0.745
**SSQP3_Q5 –** Do you find it easy to distinguish different pieces of music that you are familiar with?		-0.105	0.447
**SSQP3_Q6 –** Can you tell the difference between different sounds, for example, a car versus a bus; water boiling in a pot versus food cooking in a frypan?		-0.090	0.513
**SSQP3_Q7 –** When you listen to music, can you make out which instruments are playing?		-0.058	0.672
**SSQP3_Q8 –** When you listen to music, does it sound clear and natural?		-0.099	0.472
**SSQP3_Q9 –** Do everyday sounds that you can hear easily seem clear to you (not blurred)?		-0.204	0.139
**SSQP3_Q10 –** Do other people’s voices sound clear and natural?		-0.125	0.362
**SSQP3_Q11 –** Do everyday sounds that you hear seem to have an artificial or unnatural quality?		-0.089	0.516
**SSQP3_Q12 –** Does your own voice sound natural to you?		-0.062	0.655
**SSQP3_Q13 –** Can you easily judge another person’s mood from the sound of their voice?		-0.040	0.773
**SSQP3_Q14 –** Do you have to concentrate very much when listening to someone or something?		-0.104	0.411
**SSQP3_Q15 –** Do you have to put in a lot of effort to hear what is being said in conversation with others?		-0.233	0.087
**SSQP3_Q16 –** When you are the driver in a car can you easily hear what someone is saying who is sitting alongside you?		-0.380*	0.012*
**SSQP3_Q17 –** When you are a passenger can you easily hear what the driver is saying sitting alongside you?		0.057	0.678
**SSQP3_Q18 –** Can you easily ignore other sounds when trying to listen to something?		-0.100	0.466

Spearman coefficient

* p-value ≤ 0.05

**Figure 1:**
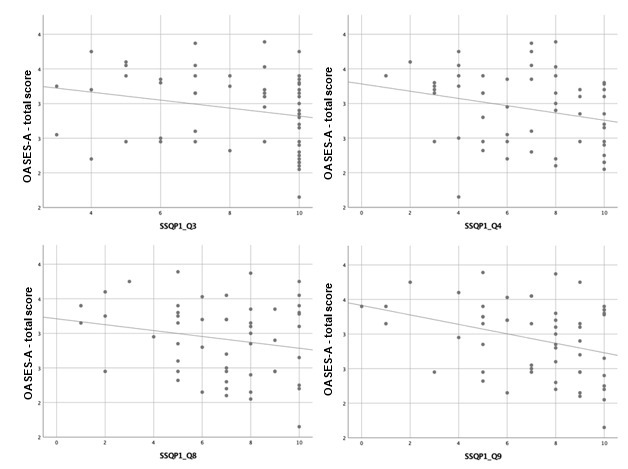
Simple scatter plot with fit line between OASES-A total score and SSQ Part 1 (questions 3, 4, 8 and 9)

**Figure 2:**
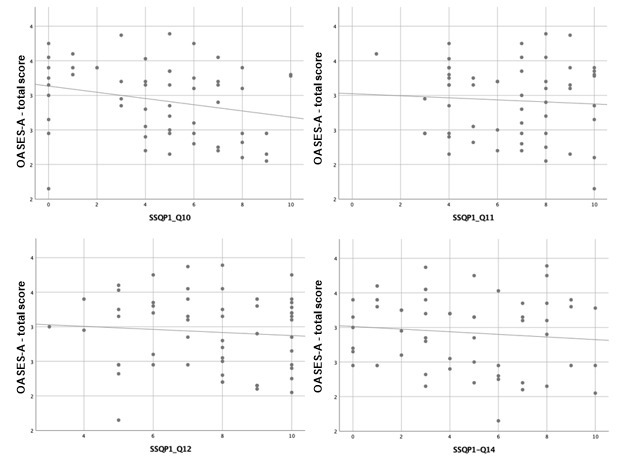
Simple scatter plot with fit line between OASES-A total score and SSQ Part 1 (questions 10, 11, 12 and 14)

**Figure 3:**
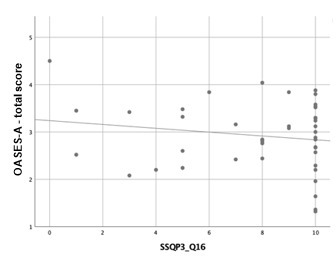
Simple scatter plot with fit line between OASES-A total score and SSQ Part 3 (question 16)

Scatterplots are presented below to better verify the magnitude and direction of the correlations found (Figures 1, 2 and 3).

## DISCUSSION

This study investigated the association between self-perceived stuttering and self-perceived hearing, speech fluency profile, and contextual factors of people with self-perceived stuttering. The results revealed an association between self-perceived stuttering and some questions in self-perceived hearing.

In the speech fluency profile, the six parameters approached in the analysis verified disruptions. Stuttering-like disfluencies occurred in greater numbers, with more than 3%, and consequently, a decreased speech speed measured with the rates of words and syllables per minute. These parameters corroborate the national and international literature^(2,4,21,24,28)^ on the characteristics of fluency in stuttering. The literature also demonstrates some important characteristics of the manifestation of stuttering related to a genetic basis that transmits susceptibility to stuttering^(28)^, age, and time of onset as positive factors for persistent stuttering^(4,21,24)^ – which agrees with the clinical data of the present study sample.

The total effect of stuttering on the quality of life of most of the sample in this study was classified as moderate to severe and their mean impact score was 3.06, despite the random sampling. This result can be explained by the fact that the adults that were interested in participating in the research, considering the self-selection method, were the ones most bothered by the disorder and, therefore, sought further help^(3)^, as most of them had already had SLH treatment.

Stuttering, with all its etiological and clinical manifestations, can lead to anxiety or limitations to effective communication, social participation, and academic or occupational performance^(12,19)^. People who stutter had at least a moderate effect on various aspects (general information, reactions to stuttering, communication in daily situations, and quality of life), according to their self-perception reported in OASES-A. This reinforces the findings on the quality of life of people who stutter^(1-3,10,12-15,18)^.

Hearing is an essential factor that interferes directly with the person’s communication capacity. Hearing sounds that surround us all the time, coming from various sources and locations, and varying over time reflect on the auditory processing skills. When a sound stands out, listeners redirect their attention, move their eyes and head toward the source, and listen carefully to understand the sound and participate in communication, especially in dialogues^(23)^. Since stuttering symptomatology includes hearing aspects, particularly those related to some auditory processing skills^(4,5,28)^, it is necessary to investigate these issues in people who stutter. Nevertheless, no research was found investigating self-perceived hearing in adults who stutter, which hindered the comparison of the findings of this study. Most pieces of research in the literature are based on behavioral auditory processing tests and assessments of people who stutter.

The association found in this study between the total classification of the effect of stuttering (obtained with OASES-A scores) and the participants’ occupations indicate that occupational aspects are related to the possible interference of stuttering on their quality of life. This finding agrees with avoidance behaviors in people who stutter, which not only reduces their social participation but also limits their effective communication and academic and occupational performance, thus affecting the quality of life^(1,2,8,10)^.

The association analysis between the total classification of the effect of stuttering and the other sociodemographic data (sex, age, educational attainment, and CCEB), clinical data (family history, stuttering history, and previous SLH therapy), and fluency profile demonstrated that these data were not related in the study sample. Hence, it can be inferred that these sociodemographic, clinical, and fluency aspects did not interfere with the self-perceived impacts of stuttering on their quality of life in this research. This finding may be due to the great fluency variability experienced by people who stutter, in whom the high occurrence of disfluencies is situational – i.e., certain communication situations may trigger a higher or lower percentage of stuttering-like disfluencies^(4,10)^, which does not necessarily change the self-assessment of the overall effect on speech. A preliminary study^(20)^ in six adults who stutter observed the variable severity of stuttering on five different days in five different speaking situations and compared the fluency assessment results with the self-perceived effect measured with OASES-A. It revealed that despite the great variance in the severity of stuttering from one day to the other, the OASES-A scores remained relatively consistent.

Furthermore, the influence of stuttering on everyday life does not depend on the frequency and type of its manifestation. Rather, suffering and its consequences depend on each subject’s singularities^(19)^, which may include intrinsic factors related to anticipatory negative thoughts about experiences and events and avoidance of certain communicative actions and/or situations involving socialization. This was reported by a study^(13)^ that indicated that the high impact of stuttering on such people’s everyday lives is associated with high levels of anxiety.

The associations found between self-perceived impacts of stuttering (OASES-A) and some items in the self-perceived hearing questionnaire (SSQ) demonstrate changes in the perception of the impacts of stuttering when compared to the perception of hearing skills in realistic communication situations. Negative correlations were specifically identified in the parts related to speech hearing and qualities of hearing – the lower the self-perceived hearing regarding the auditory skills involved in these parts, the greater the impacts of stuttering.

The moderate negative correlations in SSQ part 1 (Speech Hearing), in questions 8 (Can you have a conversation with someone when another person is speaking whose voice is the same pitch as the person you’re talking to?), 9 (Can you have a conversation with someone when another person is speaking whose voice is different in pitch from the person you’re talking to?), and 11 (You are in conversation with one person in a room where there are many other people talking. Can you follow what the person you are talking to is saying?), indicates the participants’ subjective perception of an impaired hearing skill of “speaking in speech”. Moreover, the weak negative correlations in questions 3 (You are in a group of about five people, sitting round a table. It is an otherwise quiet place. You can see everyone else in the group. Can you follow the conversation?), 4 (You are in a group of about five people in a busy restaurant. You can see everyone else in the group. Can you follow the conversation?), 10 (You are listening to someone talking to you, while at the same time trying to follow the news on TV. Can you follow what both people are saying?), and 12 (You are with a group and the conversation switches from one person to another. Can you easily follow the conversation without missing the start of what each new speaker is saying?), and in part 3 (Qualities of Hearing), question 16 (When you are the driver in a car can you easily hear what someone is saying who is sitting alongside you?) reveal the same impairment in “speaking in noise” and “listening to multiple speech flows”.

These findings are supported by the literature^(5-7,29,30)^ that reports impaired auditory processing skills in people who stutter, in comparison with people who do not stutter. The findings of this study could not be compared to other ones because this research used the participants’ self-perception of their hearing skills, and no auditory processing tests were performed. Thus, the findings are explained based on studies with clinical data and objective assessments of the auditory processing of people who stutter.

This study found disadvantage subjective perception of hearing in people who stutter, regarding the skills of speech in speech, speech in noise, and listening to multiple speech flows. This agrees with data in a study^(29)^ that verified auditory processing skills and the occurrence of the suppression effect in otoacoustic emissions in adults who stutter, comparing them with adults who do not stutter. It revealed that the investigated auditory processing skills were different in individuals who stutter and do not stutter, with greater changes in those who stutter. It also showed worse functioning of the medial olivocochlear efferent system in the individuals who stutter, indicating auditory discriminations difficulties, especially in noise.

Temporal imprecision in speech perception can cause moments of disfluency, and decreased processing skills may be related to the inability to maintain fluent speech^(6-8)^. The results of this study may indicate that the perception of the influence of stuttering on the quality of life is affected by disadvantage hearing skills as perceived by these people.

It is important to highlight that the results in this study refer only to the reference sample – i.e., adults with self-perceived stuttering, whose data were collected in a specific context. Therefore, they must be cautiously analyzed. Moreover, it cannot be inferred that self-perceived stuttering is directly related to auditory performance itself because this study did not analyze the influence of non-auditory aspects (such as the participants’ educational attainment, age, or socioeconomic level) on SSQ performance. Thus, further studies are needed to investigate the relationship between self-perceived stuttering and self-perceived hearing in various settings to better clarify how these associations and relationships take place.

This research, whose main variable was the speakers’ self-perception, made advancements concerning the relationships between the self-perceived impact of stuttering on the quality of life, self-perceived hearing, fluency profile, and contextual aspects of people who stutter in a different context from those usually found in the literature.

## CONCLUSION

The study demonstrated that self-perceived stuttering is related to some issues of self-perceived hearing, so that self-perception of auditory abilities was greater to the extent that self-perception of the impacts of stuttering on quality of life was lower.

Fluency profile was not associated with participants' self-perceived impact of stuttering on their quality of life. Occupation was the sole contextual aspect found to be associated with self-perception of stuttering in this study.

Thus, this research revealed the importance of considering the speakers’ perception of their speech to understand the factors that may be associated with such perception.
